# Spatial function of the oxidative DNA damage response in radiation induced bystander effects in intra- and inter-system of *Caenorhabditis elegans*

**DOI:** 10.18632/oncotarget.17229

**Published:** 2017-04-19

**Authors:** Qingqing Li, Jue Shi, Lianyun Chen, Furu Zhan, Hang Yuan, Jun Wang, An Xu, Lijun Wu

**Affiliations:** ^1^ Key Laboratory of Ion Beam Bioengineering, Hefei Institutes of Physical Science, Chinese Academy of Sciences, Hefei, Anhui 230031, P. R. China; ^2^ University of Science and Technology of China, Hefei, Anhui 230026, P. R. China; ^3^ Key Laboratory of Environmental Toxicology and Pollution Control Technology of Anhui Province, Hefei, Anhui 230031, P. R. China

**Keywords:** C. elegans, DDR, tissue specific RNAi, ROS, RIBE

## Abstract

Though the signaling events involved in radiation induced bystander effects (RIBE) have been investigated both *in vitro* and *in vivo*, the spatial function of these communications, especially the related signaling pathways, is not fully elucidated. In the current study, significant increases of DNA damage were clearly observed in *C. elegans* germline upon irradiation to both intra-system of posterior pharynx and inter-system of vulva, in which more severe damage, even to F1 generation worms, was shown for vulva irradiation. Spatial function assay indicated the DDR key components of *mrt-2/hus-1/cep-1/ced-4* were indispensable in germ cells for both sites irradiation, while those components in somatic cells were either not (*cep-1/ced-4*) or partially (*mrt-2/hus-1*) required to promote apoptosis. Moreover, production of reactive oxygen species (ROS) indicated by the superoxide dismutase expression and the unfolded protein response of the mitochondria was found systemically involved in the initiation of these processes for both two site irradiation. These results will give a better understanding of the RIBE mechanisms *in vivo*, and invaluable to assess the clinical relevance to radiotherapy.

## INTRODUCTION

Ionizing radiation (IR) is a well-established human mutagen and/or carcinogen known to cause tumors in various organs. On the other hand, radiation is the major therapeutic modality in the treatment of various human cancers [1]. In contrast to the radiobiology tenet in which the effects of IR are restricted to directly hit cells, radiation-induced bystander effects (RIBE) represent a paradigm shift in our understanding of the biological effects of radiation, and are of particular importance in radiation protection and linked to radiation-induced secondary carcinogenesis after radiotherapy [2]. Early reported studies of the bystander effects provided direct evidences for the production of transmissible, cell-to-cell effects between targeted and non-targeted cells individually exposed to charged particles [3, 4]. Since then, a plethora of studies *in vitro* have been performed [5]. Derives from the fact that cells respond differently in a living organism, by constantly communicating with surrounding tissues, there is a growing need for *in vivo* studies. Using partial-organ irradiation technique, Khan *et al* reported the significant molecular and cellular damage in the shielded organ parts [6], suggesting that bystander signals were communicated within the same organ/tissue. A signaling model for the induction of non-targeted responses in the “out of field” lung tissue after lower abdomen irradiation [1], indicated that bystander signals were communicated among tissues and systems, either directly or via systemic signaling. Moreover, clinical evidence of RIBE has also been found in humans in the form of radiotherapy-mediated abscopal effects, and the inflammatory signaling and the immune system have been recognized as key components of transmission in abscopal effects [7]. Though these studies demonstrated clearly that radiation damage could be transferred *in vivo*, their related signaling pathways, especially for the spatial function of radiation damage signals, are not elucidated.

*Caenorhabditis* (*C*.) *elegans* has been widely used as an *in vivo* model system in the field of radiation biology and a tool to dissect the complex signaling network. For instance, Deng *et al* discovered ceramide biogenesis and ceramide pathway were required for radiation-induced apoptosis in the germ line of *C. elegans* [8]. Using X-ray to induce DNA damage, Sendoel *et al* found HIF-1 could regulate p53-mediated apoptotic cell death through a secreted neuronal tyrosinase at a distance [9]. And, with UVB light irradiation to initiate genome instability in germ cells, activation of the ubiqutin-proteasome system (UPS) in somatic tissues and systemic stress resistance were demonstrated [10]. Besides, its transparent body, allowing for the direct visualization of specific tissues, makes it a unique model for studying precise radiobiology, such as the production and transfer of damage signals in the intra- and inter-system of *C. elegans*. A few studies have described the use of *C. elegans* for microbeam studies [11, 12]. We also reported previously that irradiation of somatic pharynx resulted in a significant induction of bystander germ cell apoptosis [13].

As a follow-up study, here, we locally irradiated either posterior pharynx or vulva of *C. elegans* as a comparison of the intra- and inter-system bystander effects and investigated the spatial function of the oxidative DNA damage response by tissue specific RNA interference. Our results showed that intra-system irradiation of vulva caused more severe damage in the germline compared to inter-system irradiation of pharynx. DNA damage response components were defined as bystander responders and function mainly in the bystander germ cells. In addition, the role of reactive oxygen species was proved to promote the bystander germ cell apoptosis.

## RESULTS

### The bystander germ cell death induced by the intra- and inter-system irradiation of *C. elegans*

**Figure 1:**
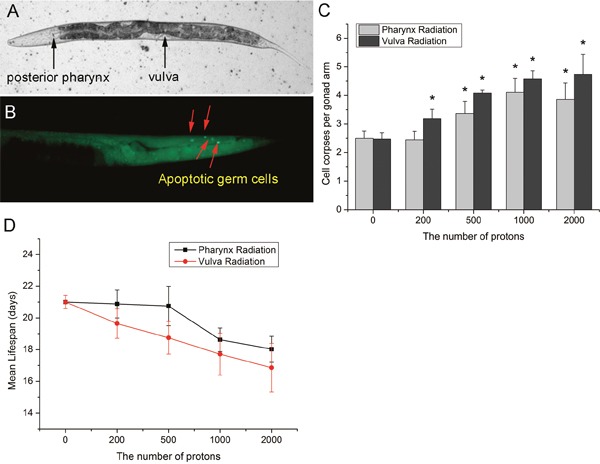
The induction of germ cell death in *C. elegans* by microbeam irradiation **(A)** The local images of worms were captured with a CCD camera. The irradiated posterior pharynx bulbs and vulvas were indicated by black arrows. **(B)** Apoptotic germ cells in gonad of *C. elegans* were indicated by red arrows. **(C)** The posterior pharynx bulbs and the vulvas of *C. elegans* at the L4 stage were irradiated respectively with the indicated numbers of protons and germ cell corpses were scored 24 hr after irradiation. **(D)** The relationship between mean lifespan and proton fluence. Data were pooled from three independent experiments. Error bars indicate ± SD. * Statistical significance at *p* < 0.05.

In the present study, the posterior pharynx and the vulva were employed to study the bystander effects induced in intra- and inter-system of *C. elegans*. Although vulva is an organ of reproductive system, it belongs to the somatic gonad, which refers to the non-germ-line component of each arm [14]. Therefore, radiation of these two sites, not only represents the bystander signaling from somatic cells to germ cells, but also compares the intra- and inter-system bystander effects. As shown in Figure 1, microbeam localized radiation to posterior pharynx bulbs and vulvas of *C. elegans* significantly increased bystander germline apoptosis in a dose-dependent manner, as revealed by AO vital staining. Compared with pharynx irradiation, irradiation to vulva could induce higher apoptotic germ cell corpses, even at low doses. Moreover, when the posterior pharynx was bombarded with 2000 particles, germ-cell apoptosis did not increase further, indicating a saturation of dose response in the inter-system bystander induction of germ cell death (Figure 1C). Since an intact germline without excessive apoptosis is necessary for longevity [15], we further investigated the mean lifespan to further ascertain the RIBE in the germline. Similar to germ cell death, both average life expectancy were reduced and the vulva irradiation led a more severe response (Figure 1D). These results demonstrated the bystander signaling in intra- and inter-system *in vivo*, and provided the evidence of system-specific radiation sensitivity in the induction of bystander responses.

### DNA double-stranded breaks (DSBs) formation in bystander germ cells

**Figure 2:**
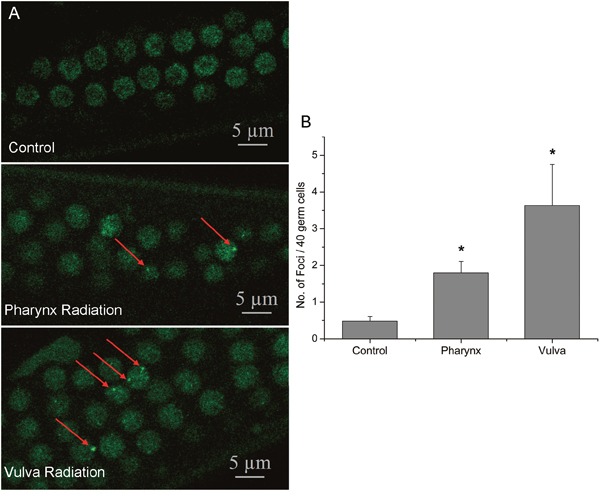
Microbeam-induced germ cell apoptosis was through DNA damage-induced germ cell death machinery **(A)** The increased DNA damage in proliferating germ cells in the worms transgenic for *hus-1::gfp*. Under a laser confocal microscope, distinct foci of HUS-1::GFP could be observed in a small number of germ cells in the mitosis region at the time point of 6 hr after irradiation, as indicated by the arrows, but not in the control worm. **(B)** The number of HUS-1::GFP foci per 40 germ cells in control and radiation groups. Data were pooled from three independent experiments. Error bars indicate ± SD. * Statistical significance at *p* < 0.05.

Radiation-induced apoptosis *in C. elegans* is directly due to DNA damage via an evolutionarily conserved checkpoint pathway [16]. To investigate the role of DNA damage in the RIBE, we examined the DNA damage in the bystander germline using the *hus-1*::*gfp* strain. In the *C. elegans*, HUS-1 is a part of the 9:1:1 complex belonging to DNA damage checkpoint protein and acts as a DNA damage sensor. Diffused HUS-1::GFP in proliferating germ nuclei relocalize and form distinct foci following DNA damage, and the foci likely represent the sites of DSBs [17]. As shown in Figure 2, both intra- and inter-system irradiation significantly caused a dramatic increase in HUS-1::GFP foci in the germline (Figure 2A) and compared with pharynx irradiation, irradiation to vulva induced more severe damage to the germline, showing that the ratio of cells containing spontaneous HUS-1::GFP foci was 0.48 ± 0.13, while the ratios of cells containing HUS-1::GFP foci increased significantly to 1.8 ± 0.31 and 3.63 ± 1.12 after the posterior pharynx and vulva were irradiated respectively. These results were consistent with apoptosis results above and further demonstrated that both two non-targeted radiation stimulated the cellular DNA damage in the distant germ line of worms and more severe DNA damage was in intra-system RIBE.

### Involvement of DNA damage-induced germ cell death machinery

**Figure 3:**
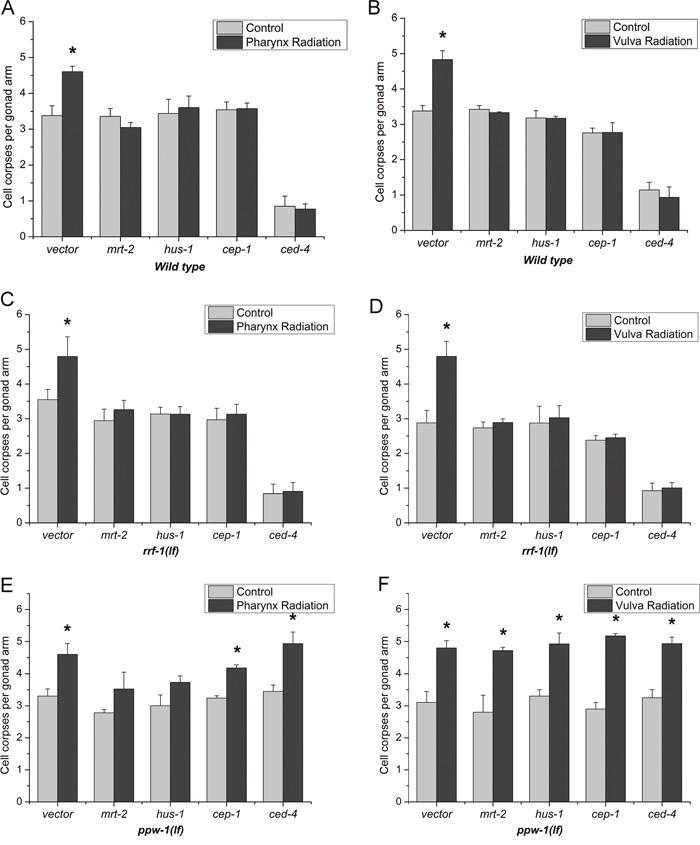
Spatial function of the DNA damage-induced germ cell death machinery Germ cell death was quantified after irradiated with 1,000 protons at the posterior pharynx bulbs and the vulvas in *wild-type*, *rrf-1(lf)* and *ppw-1(lf)* L4 worms fed bacteria producing double-stranded RNA against a control gene or *mrt-2/hus-1/cep-1/ced-4*. These data represent the average of three independent experiments. Error bars indicate ± SD. * Statistical significance at *p* < 0.05.

It has been increasingly accepted that targeted cells exposed to IR and other genotoxic agents can communicate their DNA damage response (DDR) status to bystander cells [18]. To assess whether DDR also function in the radiation induced bystander effects in *C. elegans*, we inactivated some representative genes in DNA damage response pathway by “RNAi feeding”. In *C. elegans*, MRT-2 and HUS-1 are the DNA damage checkpoint protein acting as sensors that detect the DNA damage. CEP-1 acts as a transcription factor and is able to activate DNA damage-induced apoptosis. CED-4 is required for programmed cell death [19]. As shown in Figure 3A and 3B, after the ablation of *mrt-2/hus-1/cep-1/ced-4* by RNAi, the germline apoptosis were not altered compared to the controls after both intra- and inter-system irradiation. These results were consistent with that using the loss-of-function mutants (data not shown), and revealed that these DNA damage response genes were indispensable for both two kinds of RIBE and the core apoptotic pathway was required for radiation induced bystander germ cell death.

### The spatial function of the DDR pathway

For *C. elegans*, its defined tissues can be divided into somatic cells and germ cells [20]. From the results above, we confirmed the involvement of DNA damage-induced germ cell death machinery, and the following question is how the DDR pathway took part in. Two reciprocal tissue-specific RNAi mutants *rrf-1(lg)* and *ppw-1(lg)* have been adopted [21], and the genes in the bystander signalling pathways were knocked down separately in germ cells or in somatic cells. In addition, wild-type, *rrf-1(lf)* and *ppw-1(lf)* mutants fed bacteria producing control (RNAi) had similar numbers of germ cell corpses after DNA damage.

As shown in Figure 3C and 3D, after ablation of *mrt-2/hus-1/cep-1/ced-4* in germ cells using *rrf-1(lf)* mutants, germline apoptosis were not induced compared with control for both pharynx radiation and vulva radiation, revealing that these DNA damage response genes in germ cells were indispensable for both intra- and inter- system RIBE. In contrast, selective knockdown these representative genes in somatic cells using *ppw-1(lf)* mutants showed a different performance. For pharynx radiation, selective knockdown of *mrt-2/hus-1* in the soma slightly prevented germ cell death versus knockdown of *cep-1/ced-4* had completely no inhibition on the germ cell death (Figure 3E), suggesting that MRT-2 and HUS-1 are partially required in somatic tissue to regulate germ cell death, CEP-1 and CED-4 in somatic tissue are of no use for RIBE from pharynx to germline. And for vulva radiation, specific knockdown of *mrt-2/hus-1/cep-1/ced-4* in somatic cells showed an uninhibited increase in the apoptotic germ cells (Figure 3F), indicating that *mrt-2/hus-1/cep-1/ced-4* expression in somatic tissue is not required to promote apoptosis and the DNA damage response pathway only function in the germline when the irradiation site is the reproductive system organ vulva.

### The induction of ROS in the promotion of bystander DNA damage

**Figure 4:**
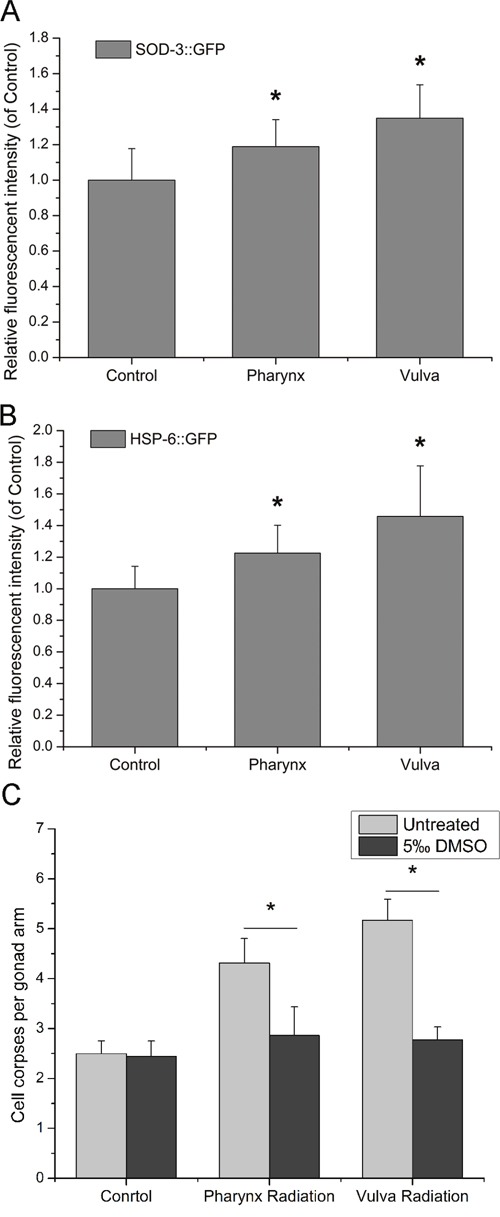
The role of ROS in the induction of bystander DNA damage **(A)** Transgenic worms CF1553 were used to determine the relative ROS production, **(B)** Transgenic worms SJ4100 were used to determine the mitochondrial folding environment, the relative fluorescence in intact worms was determined using Image-Pro Plus, version 6.0. **(C)** The induction of germ cell apoptosis by RIBE was suppressed by exposure to 5.0‰ DMSO. Data from three independent experiments were pooled. All values are shown as mean ± SD. * indicates statistical significance in comparison to controls (*P* < 0.05).

Previous data showed that DNA damage and DDR mainly took part in bystander germ cells as the responders to RIBE. Therefore, the key requirement following is to elucidate how DNA damage and DDR pathway are activated in bystander cells. It has been proposed that reactive oxygen species (ROS) in many systems function as initiator of bystander DNA damage and have connections with DDR [22]. To explore the possible role of ROS in the induction of RIBE, the systemic expression level of ROS was further demonstrated in the CF1553 and SJ4100 transgenic worms in the present study. As shown in Figure 4A and 4B, the expressions of *sod-3* and *hsp-6*, which has been considered to activate oxidative stress and the mitochondrial unfolded protein response, were induced upon irradiation to posterior pharynx bulbs and vulvas of *C. elegans* respectively. In the presence of 5‰ DMSO, the number of germ cell corpses was restored to the basal level, indicating that both intra- and inter-system RIBE could be eliminated by the free radical quencher DMSO (Figure 4C). Therefore, it can be asserted that ROS are involved in the generation or transduction of RIBE and prior to DNA damage in *C. elegans*.

### Adverse transgenerational effects in irradiated progeny

**Figure 5:**
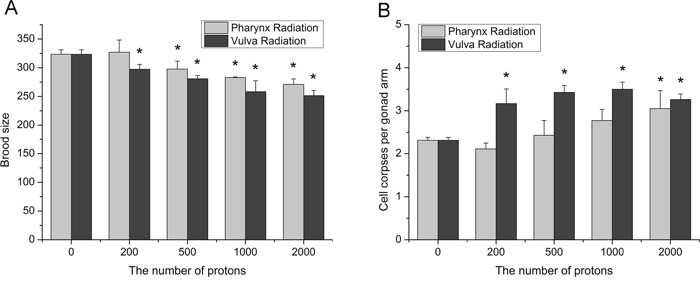
The induction of adverse inter-generational effects in irradiated progeny **(A)** The brood size was calculated by combining the number of eggs and hatched F1 larvae. **(B)** The germ cell death in F1 progeny of worms irradiated with indicated number of protons. Data were pooled from three independent experiments. Error bars indicate ± SD. * Statistical significance at *p* < 0.05.

Transgenerational effects are those occurring in the offspring following irradiation of one or both parents, and genomic instability is characterized by genetic changes in irradiated progeny [23]. To understand the transgenerational effects of radiation induced bystander effects in *C. elegans*, we took brood size and germ cell apoptosis in F1 generation as the endpoints. As shown in Figure 5A, both two kinds of RIBE decreased the progeny of irradiated worms in a dose-dependent fashion, and the adverse effect on fecundity caused by pharynx irradiation was less significant than by vulva irradiation. In comparison to the decreased fecundity, germ cell apoptosis in F1 generation increased more dramatically as a consequence to RIBE. For instance, the irradiation at vulva exhibited a drastically increase of germ cell death in irradiated progeny even at a low dose of 200 particles (Figure 5B), while the increase was conspicuous at 2,000 particles exposed locally to the posterior pharynx bulbs. These findings revealed that radiation signals could induce more severe genetic damage in intra-system RIBE and initiate genomic instability in the bystander proliferative germ line of worms.

## DISCUSSION

The manifestation of bystander effects *in vivo* has important implications for radiotherapy, by offering a possible explanation for normal tissue toxicity as well as secondary tumors in distant organs [24]. Although bystander effects have been noted in different animal tissues, the related signaling pathways within the organism are not elucidated, especially for the spatial function of radiation damage signals. In the present study, digestive organ posterior pharynx and reproductive organ vulva of the worms were adopted to compare the intra- and inter-system bystander effects. Consistent with the evidences that RIBE can occur not only within the tissue [6] but also transmit between tissues [1], significant increases of DNA damage indicated by HUS-1::GFP foci and apoptosis in germline were clearly observed upon irradiation of both sites, in which more severe damage was shown for vulva irradiation (Figure 1 and Figure 2). As RIBE *in vivo* could be regulated in a tissue-specific manner and distinct in different organs [25], and there are more intra-tissue communications than that of inter-tissue [26]. It can be speculated that RIBE signals transmit more easily within the same system, and these differences might be due to differential activation of signaling pathways [27].

A number of intracellular transducers and signaling pathways have been proposed but DNA damage response and repair processes appear to be particularly important in bystander effects [28, 29]. By systemic interference of four representative genes (*mrt-2/hus-1/cep-1/ced-4*) in DNA damage response pathway (DDR), we proved in the present study that DDR pathway was indispensable for radiation-induced bystander germ cell apoptosis both in intra- and inter-system. To further ascertain their spatial function, tissue-specific interference of these genes in either soma or germline was employed. Consistent with previous study that a DDR status could be detected in bystander cells [30], *mrt-2/hus-1/cep-1/ced-4* were found to mainly function as bystander effectors in the germline (Figure 3). Compared to DDR networks in somatic tissue are of no use for RIBE in intra-system, DNA damage checkpoint protein MRT-2 and HUS-1 are partially required in somatic tissue to regulate germ cell death for inter-system RIBE. Although the DDR signaling proteins are undetectable in the somatic cells of *C. elegans* due to transcriptional repression [31], detectable upstream DNA damage checkpoint expression in partial cells of pharynx bulbs was observed [32]. This might be the reason that MRT-2 and HUS-1 are partially required in somatic tissue when the RIBE was initiated at the posterior pharynx.

There are many inducers of RIBE that contribute to the signals transmitted to the non-targeted cells [5]. Plenty of evidences indicated that oxidative stress and the consequently derived DNA lesions function as key factors for the development of radiation-induced bystander effects [33]. To study systemic expression level of ROS, two transgenetic mutants CF1553 and SJ4100, which specifically indicated the expression of GFP-labeled mitochondrial manganese superoxide dismutase SOD-3 [34] and the unfolded protein response of the mitochondria [35] were used. The results showed that despite the increased incidence of DNA damage observed in bystander germline, the enhanced level of ROS production was observed systemically for both two site irradiation, and could be recovered by the free radical quencher DMSO, suggesting that oxidative damage played a pivotal role in the transduction of RIBE. Moreover, by comparing these two kinds of RIBE, we discovered that irradiation at vulva produced a higher level of ROS production than that at pharynx. It seems that more severe damage to DNA by vulva radiation in turn increased the secondary ROS-production through regulatory processes [36], and therefore led to a higher level of ROS production systemically.

**Figure 6:**
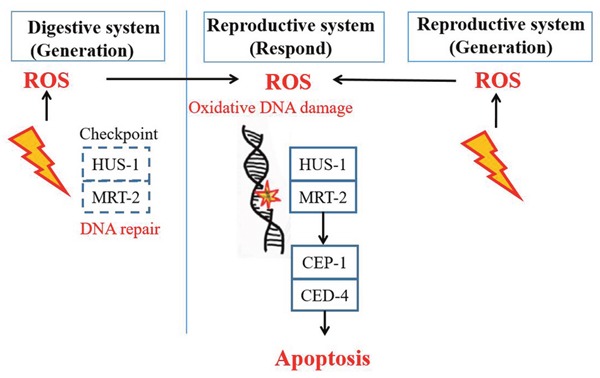
A signaling model for the induction of non-targeted cell death in the germline of *C. elegans* after intra- and inter-system irradiation

Given all the above, it can be speculated that the spatial signalling for intra- and inter-system RIBE could be initiated by ROS and activated through DDR pathway (Figure 6). Besides, the checkpoint components (HUS-1 and MRT-2) were found partially required in inter-system RIBE, but not in intra-system. More importantly, the persistence of such stressful effects could be transferred to their progeny, indicating an increasing risk of genomic instability.

## MATERIALS AND METHODS

### Worm strains and maintenance

*C. elegans* strains were maintained under standard conditions at 20°C using *Escherichia coli* OP50 as a food source [37], except when subjected to RNAi treatment. To obtain synchronized cultures, gravid hermaphrodites were lysed in an alkaline hypochlorite solution. We used these previously reported strains in this study:

Wild Type: Bristol N2,

WS1433: *hus-1(op241)* I; *unc-119(ed3)* III; *opIs34*,

NL2098: *rrf-1(pk1417)* I,

NL2550: *ppw-1(pk2505)* I,

CF1553: *muIs84* [(*pAD76*) *sod-3p::GFP + rol-6(su1006)*],

SJ4100: *zcs13*[*hsp-6::GFP*].

### Microbeam-localized irradiation of *C. elegans*

The proton microbeam facility in our laboratory (CASLIBB) delivering defined numbers of charged particles was used for the localized irradiation of *C. elegans*. The average energy range of incident protons was 2.0~3.0 MeV with a LET of 11 keV/μm and the average beam diameter on samples measured less than 10 μm using CR-39 solid detectors for 10,000 protons [38]. For microbeam irradiation, synchronized worms at the L4 stage were picked out and placed on 2% of agarose gels and then anesthetized as described previously [13]. To study the spatial function of RIBE, the posterior pharynx bulb and the vulva of *C. elegans*, which were both easily distinguishable under the integrating CCD camera and are far from the observed gonad (Figure 1A), were chose as the intra- and inter-system RIBE irradiation sites. Upon irradiation, the treated worms and their mock-controls were washed from the Mylar film with M9 buffer and allowed to recover on new NGM agar or RNAi plates for further analysis.

### Apoptosis assay

Apoptotic germ cells were measured by acridine orange (AO) vital staining as described [39]. Briefly, 200 μl of freshly diluted AO solution (75 μg/mL) was pipetted onto a plate containing at least 25 irradiated adult worms on a bacterial lawn. After 1hr of incubation in the dark, the worms were then transferred to a clean NGM plate for recovery to clear excess AO from the intestines. The worms were immobilized by sodium azide and fluorescent staining was observed under an Olympus IX71 microscope (Olympus, Tokyo, Japan).

### RNA interference

RNAi was carried out following the standard procedures [40]. Briefly, bacteria expressing doubled-stranded RNA to a specific worm gene were grown on NGM plates containing 25 μg/ml carbenicillin and 1 mM IPTG. Larvae at the L1 stage of development were placed on the RNAi-feeding bacteria plates and allowed to develop to the L4 stage. Worms were then treated with IR, allowed to recover for 24 hr on a fresh RNAi plate, and germline-apoptosis quantified as above. An L4440 vector was used as negative control in RNAi experiments, while an *unc-15* RNAi clone was included in the experiments as a positive control. Reciprocal tissue-specific RNAi mutants *rrf-1(pk1417)* and *ppw-1((pk2505)* were adopted to knock down genes separately in germ cells or somatic cells [21].

### DNA damage and ROS measurement

DNA damage in the *C. elegans* germ line was assessed using the strain *hus-1::gfp*, as described previously [17]. Worms were mounted on microscope slides in 0.2 mM Levamisole (Sigma), and the foci in a single Z stack were counted under a laser confocal microscope (LSM710 Zeiss, Germany). Approximately 40 mitotic germ cells could be observed. For ROS measurement, the transgenic strain CF1553: *muIs84*[(pAD76)*sod-3p*::GFP+*rol-6*(*su1006*)], containing the SOD-3::GFP-linked reporter, was used to visualize the expression of SOD-3. And, the transgenic strain SJ4100: *hsp-6*::*gfp*(*zcIs13*), which contains the mitochondrial misfolded protein stress reporter encoding a mitochondrial chaperone, was adopted to monitor a mitochondrial unfolded protein response. The worms were immobilized by Levamisole (Sigma) and fluorescent images were acquired using the 20X objective of an inverted microscope (Olympus IX71). The relative fluorescence was determined metrically using Image-Pro Plus, version 6.0.

### Data analysis

All values were expressed as means ± standard deviation of the means. Significant differences at the *P* < 0.05 level were tested using ANOVA followed by Dunnett's *t*-tests or two-tailed Student's *t*-tests. A *P* value of 0.05 or less between groups was considered to be significant, marked as * *P* < 0.05.

## Abbreviations

RIBE: radiation induced bystander effects
DDR: DNA damage response
ROS: reactive oxygen species
NGM: nematode growth medium
